# Increased MSX level improves biological productivity and production stability in multiple recombinant GS CHO cell lines

**DOI:** 10.1002/elsc.201900124

**Published:** 2020-01-09

**Authors:** Jun Tian, Qin He, Christopher Oliveira, Yueming Qian, Susan Egan, Jianlin Xu, Nan‐Xin Qian, Erik Langsdorf, Bethanne Warrack, Nelly Aranibar, Michael Reily, Michael Borys, Zheng Jian Li

**Affiliations:** ^1^ Biologics Process Development Global Product Development and Supply, Bristol‐Myers Squibb Company Devens MA USA; ^2^ Molecular & Cellular Science Bristol‐Myers Squibb Company Princeton NJ USA; ^3^ Drug Development and Preclinical Studies Bristol‐Myers Squibb Company Princeton NJ USA

**Keywords:** biologics manufacturing, bioprocessing, methionine sulfoximine (MSX), monoclonal antibody, specific productivity

## Abstract

Increasing cell culture productivity of recombinant proteins via process improvements is the primary focus for research groups within biologics manufacturing. Any recommendations to improve a manufacturing process obviously must be effective, but also be robust, scalable, and with product quality comparable to the original process. In this study, we report that three different GS^−/−^ CHO cell lines developed in media containing a standard concentration of the selection agent methionine sulfoximine (MSX), but then exposed to increased MSX concentrations during seed train expansion, achieved titer increases of 10–19%. This result was observed in processes already considerably optimized. Expanding the cells with a higher MSX concentration improved cell line production stability with increased culture age. Production cultures in 500‐L and 1000‐L bioreactors replicated laboratory results using 5‐L bioreactors, demonstrating process robustness and scalability. Furthermore, product quality attributes of the final drug substance using the higher MSX process were comparable with those from cells expanded in media with the standard selection MSX concentration. Subsequent mechanistic investigations confirmed that the cells were not altered at the genetic level in terms of integration profiles or gene copy number, nor transcriptional levels of glutamine synthetase, heavy chain, or light chain genes. This study provides an effective and applicable strategy to improve the productivity of therapeutic proteins for biologics manufacturing.

**Abbreviations**
CHO: Chinese hamster ovary
GCL_c_: Glutamate‐Cysteine Ligase Catalytic Subunit
GCL_m_: Glutamate‐Cysteine Ligase Modifier Subunit
GOI: gene of interest
GS: glutamine synthetase
HC: heavy chain
LC: light chain
MSX: methionine sulfoximine
VCD: viable cell density

## 1 INTRODUCTION

The size of the therapeutic biologics market and future growth potential emphasizes the importance for continued optimization of manufacturing processes. Biologics account for 17% of the total pharmaceuticals approved by the U.S. Food and Drug Administration and the European Medicines Agency in the past 20 years. This percentage increased to 38% in the past 3 years 1, 2. The individual sales for 42 of the approved biologics surpassed 1 billion U.S. dollars (USD) and eight of them topped USD 5 billion in 2016 3. Total biologics revenue is forecasted to reach approximately USD 400 billion by 2025 4, with the mAbs segment garnering sales of USD 140 billion by 2024 5. Investments into biopharmaceuticals continue to grow due to the combination of high efficacy, suitable safety profiles, and high approval rates compared to small molecule drugs 6.

Chinese hamster ovary (CHO) cells are the most prevalent system for biologics production using mammalian cells and are currently used in 70% of industrial processes for biological therapeutic production 7. Since approval of the first monoclonal antibody in 1986, manufacturing efficiency for biologics has improved tremendously. Currently protein titers over 10 g/L have become attainable using fed‐batch culture processes 7, 8, 9, 10. Nevertheless, process yield for a number of biologic manufacturing processes is capped at approximately 5 g/L 9, 10, thus there remain significant opportunities to identify process improvements to further increase yields and/or reduce manufacturing costs.

One critical measure of process yield is the cell specific productivity rate (*q*_p_) of the target protein by the clone used for manufacturing 11. Improvement of *q*_p_ can be accomplished by screening clones based on high productivity, but also by increasing the productivity of an already selected cell line through modifications at the protein or cellular level, and by process optimization.

*Q*_p_ may be affected by a variety of factors including the primary amino acid sequence of the expressed protein 12, the global cellular gene expression regulation for vesicle trafficking, endocytosis and cytoskeletal elements 13, 14, 15, 16, 17, the activities of the mammalian target of rapamycin pathway and global protein translation 18, 19, the function activity of mitochondria 8, 20 as well as the extracellular and intracellular redox environment 8, 21. Modulation of intracellular microRNA (miR) levels has been shown to successfully increase *q*_p_ by regulating cell cycle with miR‐7 22, 23, protein synthesis, secretion and transport with miR‐557 and miR‐1287 24 and mitochondrial genome‐encoded small RNA (mitosRNA‐1978) 25, and by balancing unfolded protein response (UPR) program with miR‐1287 26. These studies demonstrated the feasibility of enhancing *q*_p_ by cell line engineering.

**PRACTICAL APPLICATION**

This study provides a method to improve the productivity of industrial cell culture processes. Clones developed and selected using a standard MSX concentration can be cultured with increased MSX concentration at manufacturing scale. This results in increased titer and a mitigation of productivity losses associated with increased cell generation. The increased MSX process is also transferrable from the development laboratory to the manufacturing scale. Furthermore, this study did not identify any concerns related to the drug substance or cell line genetic stability. The increased MSX strategy exhibited no influence on critical protein quality attributes, transgene integration, gene copy number, or clone population uniformity. The effectiveness, ease of implementation, scalability, and potential absence of negative product quality or genetic stability effects make this optimization strategy valuable to process development, biologics manufacturing, and general research.

Once a cell line or clone is selected, optimization of global process strategy and cell culture media formulation may continue to increase *q*_p_ and process yield 8, 27. For example, lower culture temperature has been shown to increase *q*_p_ by stabilizing the target gene mRNA 28, or by altering cellular metabolism and decreasing cell growth 29. Increased media osmolality alone may elevate cellular nutrient and energy metabolism by upregulating several glycolytic enzymes, thereby boosting *q*_p_ 30, 31. Addition of individual chemical compounds, such as DMSO was shown to enhance *q*_p_ by upregulating chaperone proteins that facilitate protein post‐translational modification and secretion 32. Cysteine, a conventional media component, can be replaced with S‐sulfocysteine to improve stability of this nutrient. Prevention of cysteine degradation may assist modulation of the cell culture redox environment, thus resulting in higher *q*_p_ 21. Application of tyrosine‐, histidine‐, or glutamine‐containing dipeptides, substitution of glutamine with alpha‐ketoglutarate (alpha‐KG), or altering the glutamine levels in media have been reported to lower lactate and ammonia production, thus providing better pH maintenance, improved *q*_p_, and overall process yield 33, 34, 35, 36. Yeast extracts produced a beneficial effect on *q*_p_ by maintaining the mammalian target of rapamycin pathway activity and enhancing protein translation 19, 37.

Finally, the use of small molecules has been actively explored to boost *q*_p_ and process yield. Hydrocortisone 38, lithium chloride (LiCl) 39, as well as other small molecule cell cycle inhibitors 40 have been reported to increase *q*_p_ by controlling cell growth and extending culture viability. A similar strategy has been employed by altering the sodium to potassium ratio in cell culture media 41. Other reported small molecule chemical compounds include sodium butyrate and valproic acid: sodium butyrate influenced *q*_p_ by altering cellular energy metabolism 42, 43 and upregulating genes for protein trafficking steps 13; valproic acid is hypothesized to inhibit histone deacetylase, thereby enhancing the transcription of the target gene 44, 45.

An alternative strategy for *q*_p_ improvement utilizes an additive commonly procured as a raw material. Methionine sulfoximine (MSX) is a small molecule compound that serves as a selection reagent for clone generation with a glutamine synthetase (GS) gene based expression system 46, 47. The application of 25–50 µM MSX generally provides sufficient selection stringency 48. Recent host cell engineering efforts fully knocked out the endogenous GS gene (GS^−/−^) in CHO cells 49. However, these newly engineered cell lines undergo sufficient selection stringency in media with lowered MSX concentrations or in the absence of MSX entirely. Our own experimental data, coupled with recent literature reports 48, 49, 50, suggest that increasing the MSX level at the seed train stage can increase production bioreactor *q*_p_ and improve overall process yield.

Here we present a case study that increasing the MSX level from 6.25 µM at the clone selection stage to 25 µM at the seed train stage improved cellular productivity and maintained better production stability without compromising the clone genetic stability, process robustness, scalability, or product quality comparability. In addition, some mechanisms relating to the improvement in productivity by increasing the MSX concentration are discussed. The results suggest that increasing MSX to a certain level at the seed train stage is a feasible industrial process improvement strategy for biologics manufacturing.

## 2 MATERIALS AND METHODS

### 2.1 Cell culture

Three different recombinant GS^−/−^ CHO cell lines (A, B, and C) expressing different IgG monoclonal antibodies were cultured in a proprietary, chemically‐defined, and glutamine‐free medium containing 6.25, 25, or 75 µM MSX. Cell lines were cultured in shake flasks and passaged every 3–4 days into fresh media. WAVE (GE Healthcare) bioreactors or 200‐L disposable Xcellerex (GE Healthcare) bioreactors were used for seed expansion when large cell numbers were required.

Production bioreactors were run in fed‐batch mode using proprietary, chemically‐defined, glutamine‐free basal and feed media in 5‐L glass (Finesse), 500‐L Xcellerex (GE Healthcare), or 1000‐L Xcellerex (GE Healthcare) bioreactors. Viable cell density (VCD) and viability were measured using a Vi‐Cell XR (Beckman Coulter).

### 2.2 Titer

Cell culture samples were analyzed for titer on an Acquity UPLC‐H Class (Waters) with a POROS A/20, 2.1 × 30 mm column (LifeTechnologies). The mobile phase used was Dulbecco's phosphate buffered saline, pH 7.4 for binding and Dulbecco's phosphate buffered saline, pH 2.6 for elution. Protein was detected at 280 nm. Titer results have been normalized to the control peak results. The specific productivity was calculated with the method described in the literature 51.

### 2.3 Protein purification

Cell culture samples were purified by affinity chromatography using MabSelect resin (GE Healthcare). The purified samples were washed with 5 mM succinic acid, pH 5.8 and eluted with a 25 mM glycine, 25 mM succinic acid, pH 3.0 buffer. The samples were neutralized to pH 6–8 with 2 M Tris. The purified samples were used to analyze protein purity, charge variants, size variants, and N‐linked glycosylation.

### 2.4 Purity

Purified samples were analyzed for protein purity using a LabChip GXII with a Protein Express LabChip and reagent kit (Perkin Elmer).

### 2.5 Charge variants

Purified samples were analyzed for charge variants on an iCE3 (Protein Simple) with a FC Column Cartridge (Protein Simple). Samples were prepared by mixing with an ampholyte solution consisting of urea, methyl cellulose, and the appropriate Pharmalyte (GE). Charge variants were detected at 280 nm.

### 2.6 Size variants

Purified samples were analyzed for size variants using an Acquity TUV UPLC (Waters) with a BEH200 SEC, 4.6 mm i.d. × 30 mm, 1.7 µm (Waters) guard column and BEH200 SEC, 4.6 mm i.d. × 150 mm, 1.7 µm (Waters) analytical column. The mobile phase was 0.2 M potassium phosphate, 0.15 M sodium chloride, pH 6.8. Size variants were detected at 280 nm.

### 2.7 N‐Linked glycosylation

Purified samples were analyzed for N‐linked Glycosylation using GlykoPrep Rapid N‐Glycan Preparation with 2‐AB (Prozyme). N‐linked species were measured on an Acquity UPLC‐H class with fluorescence detector (Waters) detected at 330 nm excitation/420 nm emission. Mobile phases were acetonitrile and 50 mM ammonium formate, pH 4.4.

### 2.8 Immunostaining

Cell culture samples were collected during the seed train stage. The samples were stained with the method described in the literature 52. The fluorescence was measured with Guava‐8HT flow cytometer (Millipore).

### 2.9 Global metabolite analysis

Cell culture samples were collected at designated days from a fed‐batch culture. The cells were removed by centrifugation at 500 RCF for 5 min at 4°C. The resulting supernatant was then analyzed for metabolites using LC–MS and NMR spectroscopy technology 53.

### 2.10 Gene copy number analysis

Cell samples on day 8 of the production fed‐batch cultures were collected for genetic analysis. Gene copy number was determined by real time qPCR analysis. Genomic DNA isolated from frozen cell pellets containing 5 × 10^6^ cells was used as substrate in real‐time qPCR utilizing primers specific to mAb molecules. The copy number was determined by extrapolating from standard curves. A range of 320 to 1 000 000 copies of DNA containing the target genes was spiked in a background of untransfected CHO cell DNA. The standard curve for the GAPDH normalizing gene was generated using five dilutions of CHO cell DNA ranging from 0.096 to 60 ng. The untransfected CHO cell DNA and no template controls were used as negative controls. Independent qPCR analyses were performed in duplicate. For each replicate, sample reactions were run in triplicate. The reactions were assembled using the Hamilton Starlet liquid handling robot and reactions were thermal cycled on the ABI Viia7 (Applied BioSystems).

### 2.11 Southern blot analysis

The structural and integration site profiles of the target genes were determined using Southern blot analysis. Genomic DNA from frozen cell pellets containing 80 × 10^6^ cells were isolated and digested with a single enzyme from a set of enzymes that restrict the target gene expression plasmid. An amount of 10 µg of genomic DNA samples were digested using the restriction enzyme NdeI to determine the number of integration sites; 10 µg of these genomic DNA samples were digested with BamHI and NdeI in order to confirm the structure of the gene cassettes. Untransfected parental cells served as negative controls. The digested DNA was subjected to agarose gel electrophoresis and analyzed using Southern blot hybridization. The gene specific hybridization probe was amplified from the target gene expression construct and labeled with digoxigenin.

### 2.12 mRNA level analysis

The relative mRNA levels of the GS, heavy chain, light chain, Glutamate‐Cysteine Ligase Modifier Subunit (GCL_m_), or Catalytic Subunit (GCL_c_) genes were determined using qPCR. The cell pellet samples containing 5 × 10^6^ cells were collected on day 8 of production fed‐batch cultures in 5‐L, 500‐L, or 1000‐L bioreactors. The cell pellet samples were kept frozen at −80°C until analysis. mRNA was isolated from the samples with PureLink^®^ RNA Mini Kit (cat.# 12183018A; Life Technologies), and was reverse‐transcribed to cDNA with High‐Capacity RNA‐to‐cDNA™ Kit (Cat.# 4387406; Applied Biosystems). The cDNA was subject to qPCR analysis with TaqMan^®^ Gene Expression Master Mix (Cat.# 4369016; Thermo Fisher Scientific) and Applied Biosystems^®^ 7500 Real‐Time PCR Systems (Applied Biosystem). All protocols followed manufacturer's instructions. The primers used for the qPCR were for TaqMan Gene Expression Assays at Thermo Fisher Scientific and are listed in Table 1. The relative expression level for the gene of interest (GOI) was reported as the delta C_t_ (ΔC_t_), the GOI C_t_ subtracted by the C_t_ for the control gene GAPDH. The standard deviation for the delta Ct was calculated as the square root of the sum of the squares of the standard deviation for the GOI and GAPDH.

**Table 1:** Primers for mRNA Level Analysis

Genes	Primer Sequence (5′ ‐ 3′), Assay ID and Catalog Number (Cat. #)	
Heavy chain (Gamma)	Forward primer	gagcacctccgagagcac
	Reverse primer	ttgcaggtgtaggtcttcgt
	Internal oligo	agcagcgtggtgaccgtgcc
Light chain (Kappa)	Forward primer	tgtcttcatcttcccgccat
	Reverse primer	gtcctgctctgtgacactct
	Internal oligo	actgcctctgttgtgtgcctgctg
Glutamine synthetase (glutamate‐ammonia ligase; Glul)	Cat.#	4351372
	Assay ID	Cg04424042_sH
Glyceraldehyde‐3‐phosphate dehydrogenase (GAPDH)	Cat.#	4331182
	Assay ID	Cg04424038_gH
Glutamate‐cysteine Ligase, Modifier subunit (GCLm)	Cat.#	4351372
	Assay ID	Cg04497880_m1
Glutamate‐cysteine Ligase, Catalytic subunit (GCLc)	Cat.#	4351372
	Assay ID	Cg04486289_g1

## 3 RESULTS

### 3.1 High viability and suitable growth performance after transfer to expansion media with increased MSX concentration

**Figure 1:**
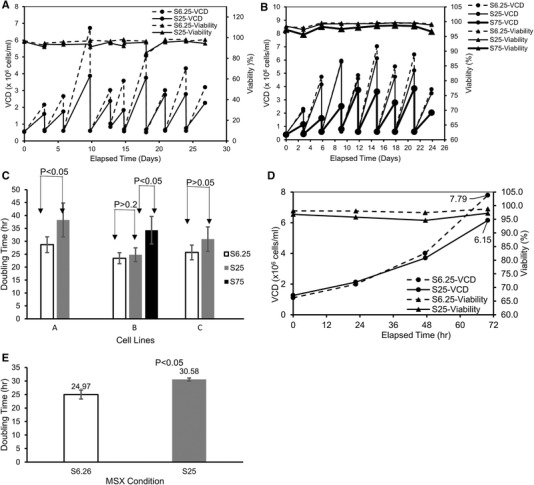
Cells were able to grow and maintain high viability in media with increased MSX concentration. Cells were cultured and expanded in the shake flasks or 50‐L WAVE bioreactors in media with different MSX concentrations (S6.25: 6.25 µM; S25: 25 µM; S75: 75 µM) for 26–30 days. Shown are VCD and viability profiles for cell lines A (A) and B (B) in shake flasks; (C) doubling time of cell lines A, B and C in shake flasks; (D) Cell line A VCD and viability in 50L WAVE; (E) doubling time of Cell line A in 50 L WAVE

Research cell banks and master cell banks were generated in expansion medium with 6.25 µM MSX concentration. To determine cell viability and growth performance at higher MSX concentrations, three GS^−/−^ CHO cell lines expressing different mAbs (cell lines A, B, and C) were directly cultured and expanded in expansion media with either 6.25 µM (S6.25), 25 µM (S25), or 75 µM (S75) MSX following the cell bank vial thaw. Growth for cell line A was slower over nine passages in S25 media (Figure 1A) resulting in an average doubling time increasing from 28.7 ± 3.0 h to 38.2 ± 6.6 h (Figure 1C). Nevertheless, cell viability remained over 95% and was comparable between the S6.25 and S25 conditions. The cells were further expanded to 50‐L WAVE bioreactors and exhibited suitable cell expansion (Figure 1D) and reasonable average doubling times of 25.0 ± 1.7 h and 30.6 ± 0.6 h for the S6.25 and S25 conditions, respectively (Figure 1E). These results demonstrated the feasibility of growing the cells in S25 media both in shake flasks and at a larger culture scale for industrial applications.

Cell line B grew at a similar rate in S6.25 and S25 media, but appreciably slower in S75 media demonstrating a MSX dose‐dependent response (Figure 1B and C). The doubling times were 23.4 ± 2.2 h, 24.8 ± 2.9 h, and 34.3 ± 6.5 h for the S6.25, S25, and S75 conditions, respectively (Figure 1C). The viability of the cells was above 95% for all the culture conditions over eight passages (Figure 1B). Cell line C behaved similarly to cell line B for cell growth and viability (data not shown and Figure 1C). These results demonstrated that the cells were able to sustain a high viability and a suitable growth rate in higher MSX media for industrial applications.

### 3.2 Increased MSX concentration during seed train stage increased productivity while maintaining protein quality attributes and clone population uniformity

To evaluate the impact of MSX concentrations on productivity and protein quality attributes, the seed cultures at the respective MSX concentrations for over 8–9 passages were then used to inoculate 5‐L production bioreactors with basal media containing no MSX. The bioreactors were fed with the respective MSX‐free feed media that was optimized for each of the different cell lines and cultured for 14 days. The VCD, viability, and metabolite concentrations were analyzed daily. Protein quality attributes were analyzed from day 14 harvest samples.

**Figure 2:**
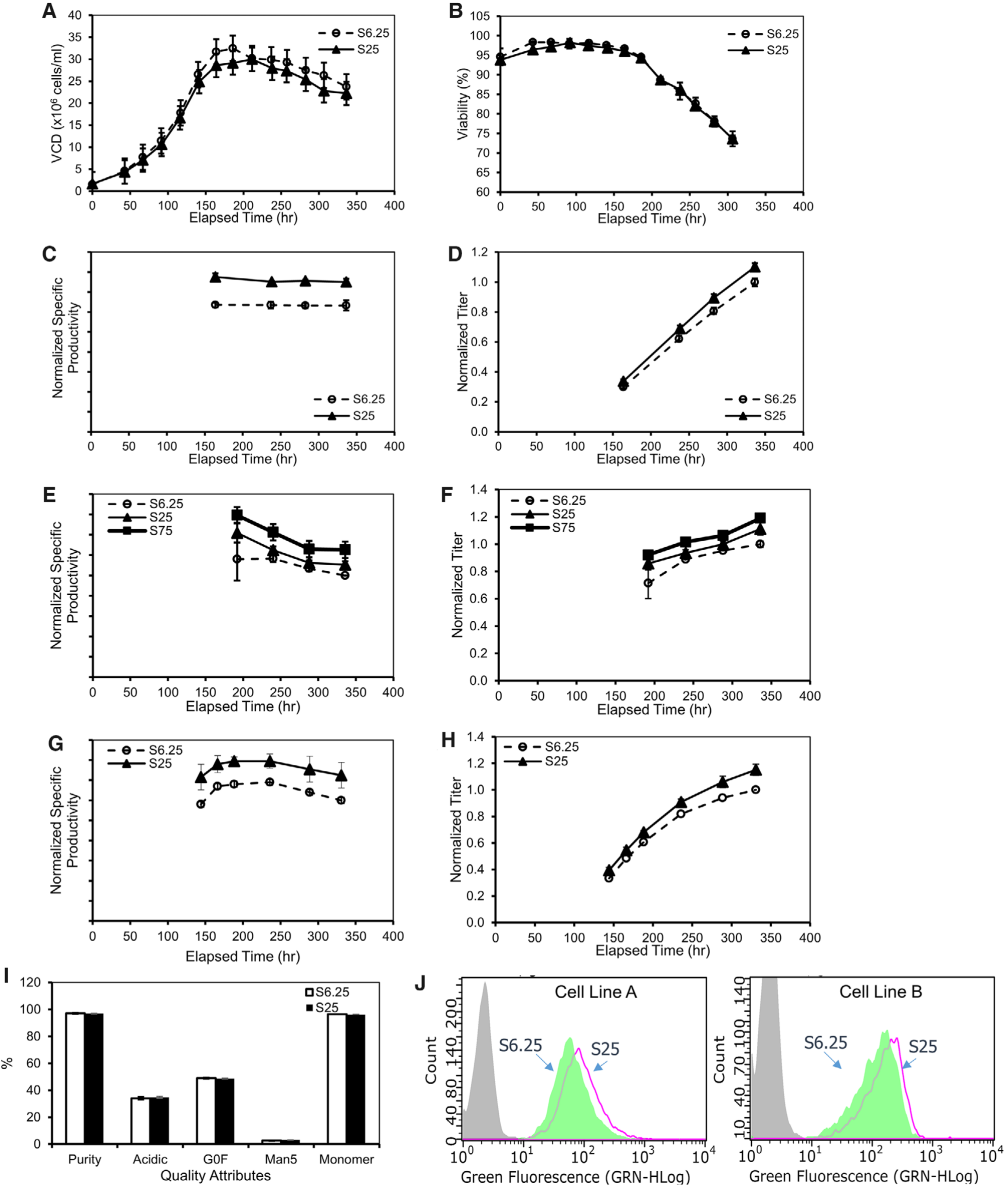
Higher MSX level boosted specific and volumetric productivity while maintaining protein quality attributes and clone population uniformity. 5‐L production bioreactors were inoculated with seeds cultured in varying concentrations of MSX (indicated as S6.25, S25, or S75) and cultured in a fed‐batch mode for 14 days. Profiles are presented for cell line A: (A) VCD, (B) viability, (C) specific productivity, (D) normalized titer; cell line B: (E) specific productivity, (F) normalized titer; and cell line C: (G) specific productivity, (H) normalized titer; and (I) protein quality attributes for cell line A; (J) histogram profiles for cell lines A and B cells immuno‐stained with anti‐IgG antibody. Grey: Isotype stained cells as negative controls; Green filled: S6.25 cells; Pink line: S25 cells

VCD and viability profiles for cell line A are shown in Figure 2A and 2B. Comparable production growth characteristics were observed for the cells expanded with different MSX concentrations. The S25 condition exhibited slightly lower peak VCD (30.0 ± 0.5 × 10^6^ cells/mL) compared to that of the S6.25 (32.5 ± 0.0 × 10^6^ cells/mL) condition. The viability profiles for cell line A were nearly identical for the S6.25 or S25 conditions (Figure 2B). Interestingly, the S25 cultures led to 19% increase in normalized specific productivity as compared to the S6.25 condition (Figure 2C). The normalized titer of the S25 cultures also increased by 10% (1.10 ± 0.02 compared to the S6.25 cultures 1.00 ± 0.03) (Figure 2D).

Normalized specific productivities for cell lines B and C also increased with increased MSX concentrations in the expansion media. The normalized specific productivity and titer for cell line B increased by 26% and 19%, respectively, with S75 expansion media compared to the cells from the S6.25 condition (Figures 2E and 2F). The normalized specific productivity and titer for cell line C increased 21% and 15%, respectively, using S25 expansion media as compared to cells cultured in the S6.25 medium (Figure 2G and 2H). These results demonstrated that higher MSX levels in the expansion media increased the specific productivity and normalized titer for the three cell lines tested.

Furthermore, we evaluated the effects of the MSX concentrations on protein quality attributes. Figure 2I summarizes the main protein quality attributes (PQAs) for the antibody produced by cell line A for the S6.25 and S25 conditions. No significant differences were observed in protein size variants (percent purity by Caliper NR or percent monomer by SEC‐HPLC), charge variants (percent acidic), or N‐linked glycosylation (percent G0F or Man5) (Figure 2I). Comparable protein quality attributes were similarly observed for cell lines B and C expanded in media with the different MSX concentrations (data not shown). These results suggest that the higher MSX concentrations did not affect the protein quality attributes for the three cell lines studied.

Importantly, immunostaining against IgG showed a similar fluorescence profile for both cell lines A and B cultured in either S6.25 or S25 media (Figure 2J) suggesting the cells remained as a uniform population when they were cultured in higher MSX concentration expansion media. The fluorescence intensity slightly increased when comparing S25 to S6.25 cultures, likely resulting from increases in intracellular IgG expression levels.

### 3.3 Increased MSX seed train process exhibited process robustness and scalability

**Figure 3:**
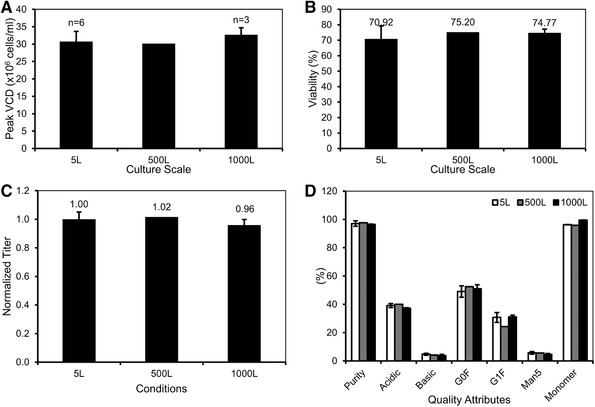
25 µM MSX strategy exhibited process robustness and scalability. The 5‐L, 500‐L, and 1000‐L production bioreactors were inoculated with cell line A S25 cells and cultured for 14 days in a fed‐batch mode. Process performance is presented for (A) peak VCD, (B) final viability, (C) normalized titer, and (D) protein quality attributes

Next, we evaluated whether the processes with increased MSX concentration in the expansion media could successfully scale‐up to manufacturing‐scale. Cell line A was directly thawed in S25 expansion media, expanded in shake flasks and WAVE bioreactors with the same S25 expansion media, and inoculated to production reactors without MSX at 5‐L bench scale, 500‐L pilot scale, or the 1000‐L scale. Peak VCD was comparable for the different culture scales: 30.7 ± 2.9 × 10^6^ cells/mL for 5‐L, 30.2 × 10^6^ cells/mL for 500‐L, and 32.7 ± 2.0 × 10^6^ cells/mL for 1000‐L cultures (Figure 3A). Harvest viability and normalized titer were also comparable among the different culture scales (Figure 3B and 3C). Protein quality attributes including size variants, charge variants, and N‐linked glycosylation were highly consistent among the different scales (Figure 3D). Cell lines B and C also exhibited comparable production performance at scales with S25 expansion cultures (data not shown). The results showed that the processes with increased MSX seed trains were robust and scalable.

### 3.4 High MSX expansion media maintained better production stability and similar genetic stability

**Figure 4:**
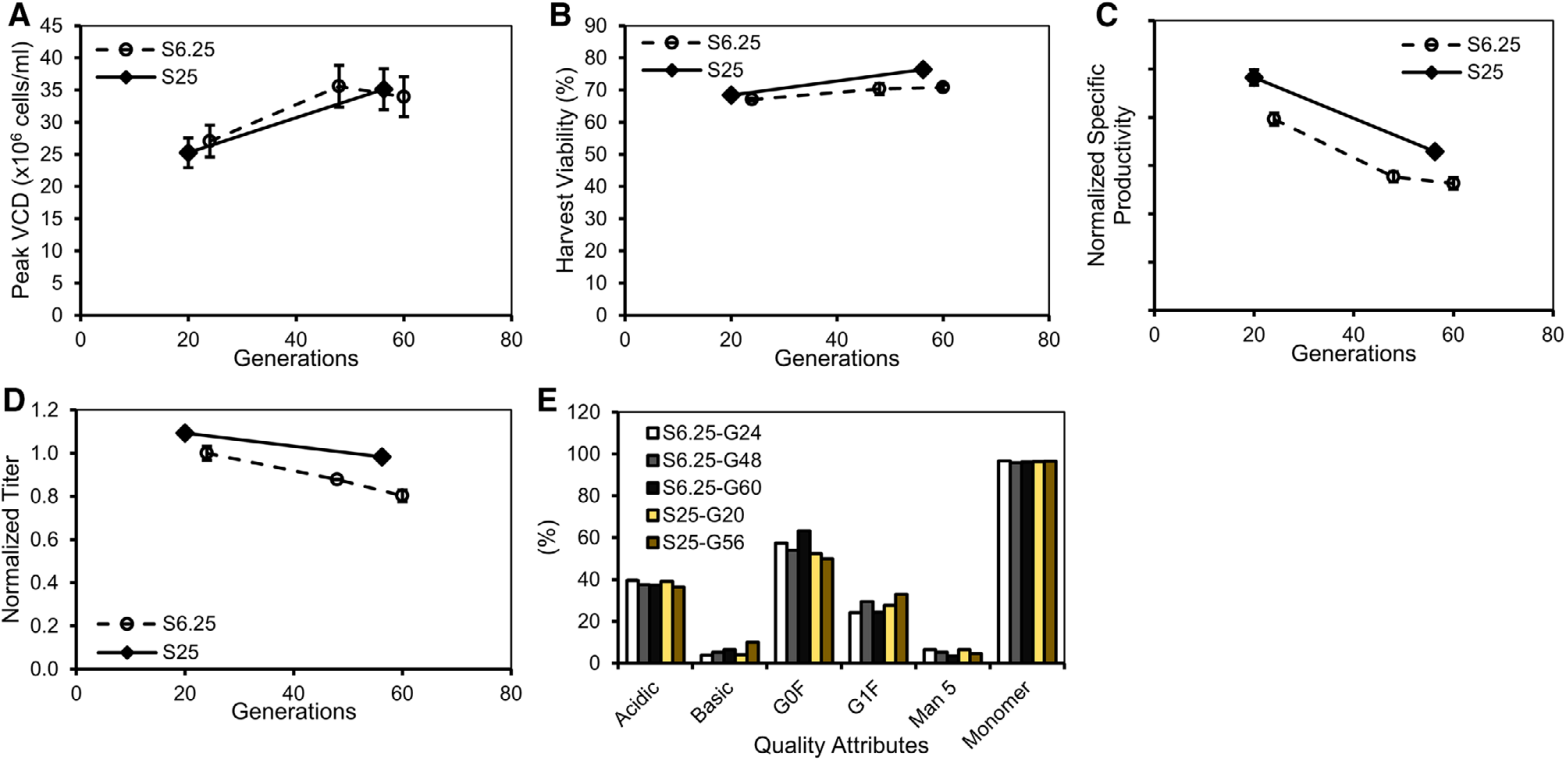
25 µM MSX maintained better production stability. 5‐L production bioreactors were inoculated with cell line A cells at different cell ages expanded with S6.25 or S25 media. The cells were cultured for 14 days with a fed‐batch mode. Presented are (A) peak VCD, (B) harvest viability, (C) specific productivity, (D) normalized titer, and (E) protein quality attributes

The beneficial effects of S25 expansion condition on titer improvement were repeated at different scales. S25 was also the most practical concentration to which MSX could change at our commercial process development stage. We thus selected S25 for further evaluation to determine the impact on the clone stability as productivity and genetic stability are important process attributes. Cell line A was subcultured to generation 60 in either S6.25 or S25 expansion media. Cells at different ages (generations) were then banked, re‐thawed, expanded for eight additional passages, and inoculated for production cultures in 5‐L bioreactors. Cell pellets on culture day 8 were collected for genetic stability analysis. Peak VCD for the S25 condition increased from 25.3 ± 2.3 × 10^6^ cells/mL to 35.1 ± 3.2 × 10^6^ cells/mL from cells at generation 20 to generation 56, and the peak VCD for the S6.25 condition increased similarly to that of the S25 condition when the cells were aged to generation 60 (Figure 4A). Harvest viability stabilized around 70% regardless of the cell age or MSX concentration (Figure 4B). The *q*_p_ for the S25 condition was 22% higher at generation 20 than that of the S6.25 condition at generation 24. The *q*_p_ increase was maintained at a similar level at 25% when the cells were aged to generations 56 (for S25) to 60 (for S6.25) (Figure 4C). Normalized titer decreased 10% by S25 cells aged from generations 20 to 56 (Figure 4D). However, the normalized titer for the S6.25 cells dropped 20% (Figure 4D). Linear regression showed that the S6.25 cells decreased productivity at a rate of 0.0054 unit/generation, 80% faster than the rate of decrease of the S25 cells at a 0.003 unit/generation (Figure 4D). The data suggests that the S25 cells maintained better production stability. Nevertheless, product quality attributes of acidic variants, size variants, and N‐glycan profiles were comparable regardless of the cell age or MSX concentrations (Figure 4E).

**Figure 5:**
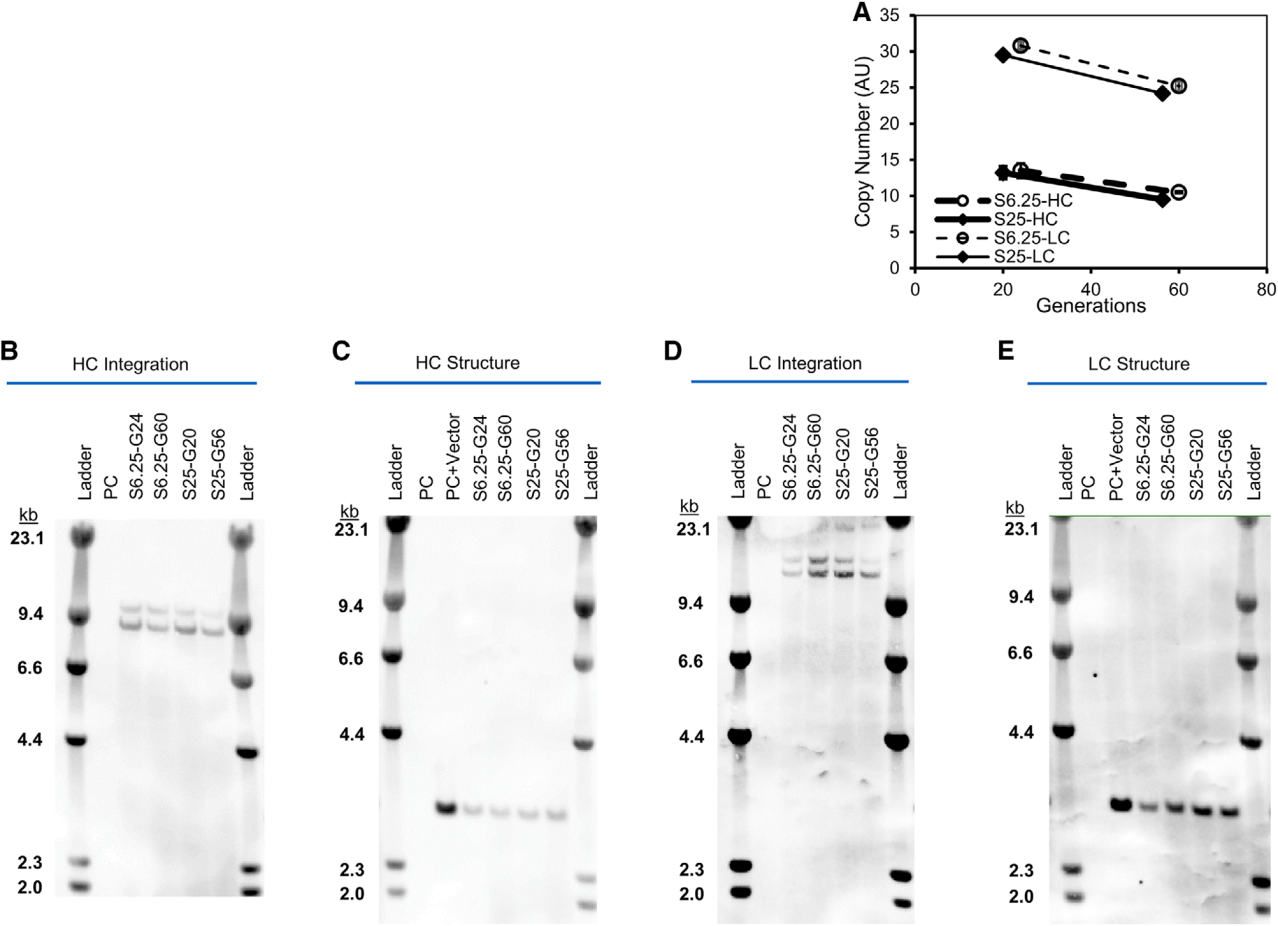
High MSX maintained similar genetic stability. Cell samples were collected from cultures presented in Figure 4 and were subject to genetic analysis. Shown are (A) gene copy numbers, (B) heavy chain (HC) integration profile, (C) heavy chain structure profile; (D) light chain (LC) integration profile, and (E) light chain structure profile. PC: un‐transfected parental cells

Finally, the antibody gene copy numbers slightly decreased in a similar manner with age for both S6.25 and S25 cells (Figure 5A). To evaluate the stability of the transgenes, Southern blot analyses were conducted using the genomic DNA isolated from the cells collected on culture day 8. A restriction enzyme was applied to the genomic DNA to evaluate the integration profiles with one site cut on the transgene and the other cut on the host cell genomic DNA. A similar strategy was employed to evaluate the structural integrity of the transgenes by using two restriction enzymes to cut outside of the transgenes. Figure 5B and 5D presented the integration profiles for the heavy chain (HC) and light chain (LC) genes, respectively. The band profiles were similar for either HC or LC genes regardless of the cell age or MSX concentration suggesting the transgenes did not shift their integration locations during extended culture or with the increase of MSX concentrations. Similarly, Figure 5C and 5E showed that the structure of the transgenes for either HC or LC remained intact at different cell age with different MSX concentrations. The data suggested that a higher MSX concentration did not impact the transgene integration profiles or structure integrity.

### 3.5 S25 expansion media exerted no impact on target gene transcription

**Figure 6:**
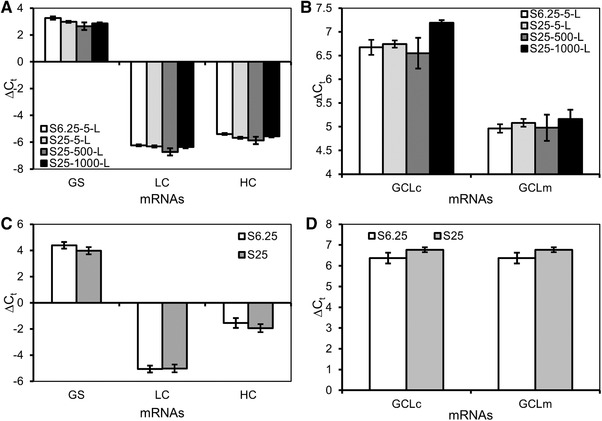
High MSX maintained similar transcription levels for GS, LC, HC, GCL_c_, and GCL_m_ genes. The production bioreactors were inoculated with cell line A and B cells expanded in S6.25 or S25 media. The production bioreactors (no MSX) were fed and cultured for 14 days. The cell samples were collected on day 8 and were analyzed for (A) Cell line A relative GS, HC and LC mRNA levels in 5‐L, 500‐L and 1000‐L production bioreactors, (B) Cell line A relative GCL_c_ and GCL_m_ mRNA levels in 5‐L, 500‐L and 1000‐L production bioreactors, (C) Cell line B relative GS, HC and LC mRNA levels in 5‐L production bioreactors, and (D) Cell line B relative GCL_c_ and GCL_m_ mRNA levels in 5‐L production bioreactors. GS: Glutamine synthetase; HC: mAb heavy chain; LC: mAb light chain; GCL_m_: Glutamate‐cysteine ligase modifier subunit; GCL_c_: Glutamate‐cysteine ligase catalytic subunit

To explore the mechanism for the improved productivity resulting from the higher MSX concentration, the relative transcription levels of GS, IgG HC and LC were examined by qPCR. For cell line A, the relative GS transcription levels (ΔC_t_) were 2.98 ± 0.08 and 3.25 ± 0.13 cycles for S25 and S6.25 in 5‐L cultures, respectively; increase in culture scale to 500‐L or 1000‐L resulted in similar transcription levels (Figure 6A). Similarly, the relative transcription levels of the HC and LC genes were also comparable between the S25 and S6.25 conditions (Figure 6A). A recent study proposed that higher MSX level increased Glutamate‐Cysteine Ligase Modifier Subunit (GCL_m_) and Catalytic Subunit (GCL_c_) levels, two genes involved in glutathione metabolism 48. However, the relative transcription levels of GCL_m_ and GCL_c_ genes in our study were not obviously different with S6.25 or S25 cultures (Figure 6B). Cell line B behaved similarly to cell line A for target gene transcriptions (Figure 6C and 6D). These results suggest that S25 media did not dramatically regulate the transcription levels of GS, HC, LC, GCL_m_, and GCL_c_ genes for the two cell lines studied.

## 4 DISCUSSION

To the best of our knowledge, the strategy of increasing the MSX concentration in expansion media for an established clone has not yet been described for an industrial manufacturing process. In this study, we reported data for three GS^−/−^ CHO mAb producing cell lines developed in media with 6.25 µM MSX underwent *q*_p_ increases of 11‐26% and overall process titer increases of 10‐19%, after expansion in seed train media with increased MSX concentrations of 25–75 µM. Furthermore, drug substance quality attributes and cell line genetic stability were comparable to control cells expanded in media with 6.25 µM MSX. Expanding the cells with higher MSX improved cell line production stability with increased cell age. Cultures scaled‐up to 500‐L or 1000‐L bioreactors replicated the results from bench scale 5‐L bioreactors, demonstrating the scalability of the process. This study provides an effective and practical strategy to improve the productivity of therapeutic monoclonal antibodies for biologics manufacturing.

Titer improvement continues to be a focus, but also a challenge for biologics process development 7. Often, lengthy process development cycles spanning several years may be required to achieve a mature manufacturing process 8, 54. Process comparability is always a practical challenge when implementing process changes as even subtle product quality attribute differences may perplex efforts for potential process improvements. Here, we presented a strategy for titer improvement through a simple process change of increasing the MSX concentration in the seed train media. Three cell lines selected with 6.25 µM MSX media were cultured in media containing increased MSX concentrations at 25 or 75 µM. The results showed that the *q*_p_ was improved by 11–26% and volumetric titer by 10–19% in the production cultures. Literature reports also showed that protein titer was improved by up to 100% when the cells were selected from MSX‐free media and subsequently cultured in higher MSX media at 25 µM 49. Another report 48 showed that both *q*_p_ and titer were improved by approximately 100% when cells were cultured in 75 µM MSX compared to those cultured in MSX‐free media. These cell lines were developed in a high MSX concentration of 50 µM, which differs from our strategy where cells were only exposed to 6.25 µM MSX during the clone development stage. For the first time, we also report that the product quality attributes including charge variants, size variants, as well as N‐linked glycosylation profiles were comparable among the production cultures with the seed train expanded in media containing different MSX levels. The processes with 25 µM MSX were scaled up to 500‐L and 1000‐L bioreactors and produced similar protein titer and quality attributes to lab scale 5‐L bioreactors. These results were consistently observed in dozens of 5‐L bioreactor experiments and multiple 1000‐L runs demonstrating the robustness and scalability of this process strategy. Taken together, increasing seed train MSX concentration to 25 µM can be an effective strategy to improve *q*_p_ and volumetric productivity for an industrial manufacturing process.

We found that the increased MSX concentration did not significantly impact cell viability, even though it increased the doubling time for the three GS^−/−^ CHO cell seed cultures. The impact of higher MSX concentration on the doubling time was clone and MSX dose dependent. Achtien et al 49 observed that eight out of nine clones exhibited significant viability decrease to 50–70% within 10–15 days after cultured in 25 µM MSX media. The viability recovered to over 90% after an additional 2–3 passages in 25 µM MSX media. One of the nine clones, however, did not experience a viability decrease in higher MSX media. Notably, the clones examined in the Achtien et al study were developed in MSX‐free media. In our study, the three cell lines evaluated were generated in 6.25 µM MSX media. The cells indeed exhibited greater doubling time or slower cell growth which suggests a certain level of stress for the cells when transferred from 6.25 µM MSX media to 25 µM or 75 µM MSX media. Nevertheless, the cells did not go through significant viability reduction, indicating no major cell population selection occurred during this adaptation process. Our study with several other antibody‐expressing cell lines had similar results (data not shown). Compared to Achtien's study, our data suggested that cells may experience greater stress resulting in a more significant viability decline when developed in MSX‐free media and transferred to higher 25 µM MSX media, compared to our system where cells were already exposed to a certain level MSX (e.g., 6.25 µM) prior to being transferred to increased MSX levels. It is also possible that different proprietary media formulations used for the two studies may have also influenced the cells’ capability of dealing with the stress from the increased MSX media.

Cell line clonality and stability are of paramount importance for biologics manufacturing processes. Indeed, gradually culturing cells in extremely high levels of MSX (e.g., 100–1000 µM) may lead to target gene amplification and may present risks to cell clonality and stability 55. Our study focused on a routinely used MSX concentration by industry peers, 25 µM, which is several fold or an order of magnitude lower than the extreme concentrations 55. In this study, the clonality of the studied cell lines was confirmed with real‐time imaging when these were developed in 6.25 µM MSX media. After the cells were cultured in 25 µM MSX media, immunostaining of intracellular IgG followed by flow cytometry analysis demonstrated that only one single cell population was present and the population distributions were similar between 6.25 µM MSX and 25 µM MSX cultures (Figure 2J). The mean fluorescent intensity was slightly higher for the 25 µM MSX culture which is consistent with higher *q*_p_ and volumetric productivity observed for the condition. Studies have shown that unstable clones, generated using either DHFR or GS selection system, developed into two or more subpopulations during a long‐term culture 50, 52. The cell lines utilized in our study, however, only presented one single cell population after extended culture up to 60 generations, suggesting that the 25 µM MSX media maintained the clone uniformity and population stability. DNA sequencing and protein peptide mapping also confirmed the absence of mutation or sequence variance for the target proteins when the cells were cultured with an elevated MSX concentration of 25 µM (data not shown). Genetic analysis confirmed that the integrated gene copy number, the target gene integration profile, the structural integrity of the transgenes, and the integrity of the mRNA transcripts of the transgenes all remained comparable to the 6.25 µM MSX cells. This genetic profile was maintained for 25 passages or 60 generations, which was sufficient for an industrial manufacturing process. Notably, when the cells were aged to 60 generations, the productivity of the 25 µM MSX cells decreased less than that of 6.25 µM MSX cells and all major PQAs tested were also preserved. Collectively, these results suggested that the cells maintained their identity (target protein sequence), clonal phenotype, as well as stability when they were cultured in 25 µM MSX media.

The mechanism of increased productivity at higher MSX concentrations has been studied but not fully understood. One hypothesis may be related to the amplification of the target gene that increases the gene copy number and thus productivity, as a higher gene copy number was observed when the clones were developed in media containing 200–1000 µM MSX compared to that in 25 µM MSX media 55. However, the gene amplification was not obvious when lower level of MSX was used. Achtien et al 49 reported that 8 out of 9 clones did not exhibit obvious gene amplification when the cells were transferred from MSX‐free media to 25 µM MSX media. One out of the nine clones did display an increase in gene copy numbers for GS and HC genes. Our analysis showed that both HC and LC gene copy numbers were comparable for cells cultured in either 6.25 µM or 25 µM MSX media. These results suggested that this productivity increase may be independent of gene copy numbers in these clones.

Another hypothesis may involve the increased mRNA transcription for target genes, as antibody productivity is correlated to the HC and LC mRNA transcripts levels 56. The increased MSX level may exert greater inhibition of GS protein, forcing cells to overexpress the GS gene thus compensating for the inhibited GS protein due to higher MSX doses. The transcription for HC and LC genes may also become enhanced given the GS and HC and LC genes co‐localize in the same region on host cell chromosomes. However, qPCR measurement showed that GS, HC, and LC mRNA levels did not markedly change when the cells were cultured in 25 µM MSX media. This result is consistent with Feary's observations 48. The Feary study proposed an alternative mechanism that higher MSX level increased GCL_m_ and GCL_c_ levels 48, two genes involved in glutathione metabolism. However, the upregulated transcription of GCL_m_ and GCL_c_ was only obvious when 50 µM or 75 µM MSX media were used, and was not obviously observed with 25 µM MSX cultures. Consistent with this report, we found that 25 µM MSX did not significantly increase the GCL_c_ and GCL_m_ mRNA levels. Taken together, the *q*_p_ and volumetric productivity increases by 25 µM MSX cannot be explained by upregulation in target transgenes, GCL_c_ or GCL_m_ gene transcripts for the two cell lines in this study.

**Figure 7:**
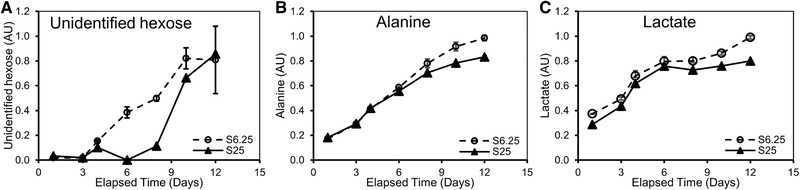
25 µM MSX altered cellular metabolic profiles. The 5‐L production bioreactors were inoculated with cells from line A cells expanded in S6.25 or S25 media. The production 5‐L bioreactors were fed and cultured for 14 days. The supernatant samples were collected at designated time points and analyzed for metabolites. Presented are profiles for (A) unidentified hexose, (B) alanine, (C) lactate

We performed global metabolic profiling and examined 120 metabolites for cell line A culture supernatants by LC–MS and NMR 53. Consistent with a recent report 50, our results showed that only a few metabolites (Figure 7) exhibited obvious differences for the 25 µM MSX condition compared to the control 6.25 µM MSX culture. Our data showed that the level of an unidentified hexose was lower for the 25 µM MSX condition, suggesting less glucose was metabolized through the pentose phosphate pathway (PPP) and shunted toward 5‐carbon sugar and other 6‐carbon sugar synthesis. Ribose 5‐phosphate was an intermediate metabolite in the PPP and served as an important precursor for nucleotide synthesis. Although we did not generate data for ribose 5‐phosphate levels, a lower level of hexose indirectly indicated a lower PPP activity, which might be related to a lower need for DNA synthesis under this condition. This correlated well with a slower cell growth in this cell line. In addition, alanine and lactate concentrations were consistently lower for the 25 µM MSX condition, indicating more glucose flowed toward the tricarboxylic acid cycle, a more efficient cellular energy metabolism. Indeed, boosted energy metabolism is of crucial importance for improving cellular *q*_p_ and productivity 42, 43. Since the metabolic profiling was only performed on cell line A, further study is warranted to determine if the results can be generalized to other cell lines.

In conclusion, we have demonstrated evidence that increasing MSX concentration after clone selection at 6.25 µM MSX improved productivity while maintaining protein quality attributes and cell line stability for three different mAb expressing cell lines. Our study provided an effective strategy to increase specific productivity and volumetric titer for therapeutic protein manufacturing processes.

## CONFLICT OF INTEREST

The authors have declared no conflict of interest.
